# Seroprevalence and risk factors of *Toxoplasma gondii* infection in sheep and goats slaughtered for human consumption in Central Ethiopia

**DOI:** 10.1186/1756-0500-7-696

**Published:** 2014-10-07

**Authors:** Endrias Zewdu Gebremedhin, Mukarim Abdurahaman, Tsehaye Hadush, Tesfaye Sisay Tessema

**Affiliations:** Department of Veterinary Laboratory Technology, Faculty of Agriculture and Veterinary Sciences Ambo University, P.O.Box 19, Ambo, Ethiopia; College of Veterinary Medicine and Agriculture, Jimma University, P.O.Box 307, Jimma, Ethiopia; College of Veterinary Medicine, Samera University, P.O.Box 132, Samera, Ethiopia; Institute of Biotechnology, College of Natural and computational Sciences, Addis Ababa University, P.O. Box 1176, Addis Ababa, Ethiopia

**Keywords:** DAT, Sheep, Goat, *Toxoplasma gondii*, Seroprevalence, Central Ethiopia

## Abstract

**Background:**

Toxoplasmosis is one of the most common parasitic zoonoses worldwide. Humans get infections with *T. gondii* after ingesting raw or undercooked meat or oocysts via contaminated soil, food or water; or congenitally by transplacental transmission of tachyzoites. The objectives of the present study were to estimate the seroprevalence and assess risk factors for *T. gondii* infection in sheep and goats slaughtered for human consumption in Central Ethiopia.

**Methods:**

A cross-sectional study was carried out from September, 2011 to November, 2012 in randomly selected small ruminants (n = 628). Direct Agglutination Test (DAT) was used to detect IgG antibodies specific to *T. gondii*. A titer of 1: 40 or 1: 4000 or both was considered indicative of *T. gondii* exposure. Logistic regression was used to assess potential risk factors.

**Results:**

An overall seroprevalence of 17.68% (111/628) (95% confidence interval [CI]:14.77 − 20.89) was detected. Twenty percent (61/305) seroprevalence (95% CI: 15.6 − 624.94) in sheep was found with a reciprocal end titers of 60 in fourteen, 180 in three, 540 in two, 1620 in five, 6000 in nine, 18000 in six, 54000 in eleven and 162000 in eleven. Similarly, seroprevalence of 15.48% (50/323) (95% CI:11.71 − 19.89) in goats was found with a reciprocal end titers of 60 in eighteen, 180 in five, 540 in three, 1620 in seven, 6000 in four, 18000 in four, 54000 in five and 162000 in four. Multivariable logistic regression analysis showed that the risk of *T. gondii* infection was significantly higher in adult sheep (adjusted Odd ratio (aOR) = 2.02, 95% CI: 1.10 − 3.70; *P* = 0.023) than in young sheep and in sheep sampled during the dry season (aOR = 4.19, 95% CI: 1.55 − 11.33, *P* = 0.005) than those sampled during wet season.

**Conclusions:**

The seroprevalence of *T. gondii* infection in small ruminants slaughtered for human consumption in Central Ethiopia is high. Age and season are significant predictors of seropositivity in sheep. The study highlighted the importance of meat of small ruminants as a potential source of infection for humans. Prevention of the spread of the disease through farm biosecurity measures is essential.

## Background

Toxoplasmosis is one of the most common parasitic zoonoses world-wide caused by *Toxoplasma gondii,* which establishes long-lasting infections in humans and animals [1, 2]. *Toxoplasma gondii* is intracellular Apicomplexan protozoan capable of infecting almost any cell type, making it one of the most ‘successful’ protozoan parasites on earth. Felids are the definitive hosts for *T. gondii* and warm-blooded species, including humans, serve as intermediate hosts [1]. Humans get infections with *T. gondii* after ingesting raw or undercooked meat, by ingesting cat-shed oocysts via contaminated soil, food or water; or congenitally by transplacental transmission of tachyzoites. However, the clinical disease is seen only in few cases [1, 3] with serious consequences in pregnant women and immunocompromised patients [1, 4]. *T. gondii* is also recognized as a major cause of reproductive failure in sheep, goats and pigs [1, 4, 5]. Recent reports show increasing number of evidences linking *T. gondii* infection with schizophrenia [6], epilepsy [7] and traffic accidents [8]. If toxoplasmosis play etiological role or attributed to the aforementioned problems then the global burden of toxoplasmosis is likely to be much higher [3] together with the life-long health care costs of congenitally infected babies. However, toxoplasmosis is still a neglected and often unreported disease despite causing a considerable global burden of ill health in humans and having a substantial financial burden on livestock industries [3].

Antibodies to *T. gondii* have been found worldwide in animals and humans [1]. Infection rate in meat-producing animals varies widely both within and between countries and is significantly higher in outdoor or extensively managed than indoor kept animals. Among livestock, pigs, sheep and goats have the highest rates of chronic *T. gondii* infection [4]. Ethiopia is among countries of the world where high occurrence of serologic evidence of *T. gondii* infection in humans [4, 9], sheep, goats, cattle [10–15], cats [16] and chickens [17] have been documented from different parts of the country. Seroepidemiological assessment of infection rates due to *T. gondii,* coupled with measures taken to reduce infection at farm level through hygienic farm management practice, is best approach to reduce the risk of *T. gondii* infection for consumers [18]. Among the serological tests, Modified Agglutination Test (MAT) is the major recommended test for diagnosis of *T. gondii* in several animals and humans with the highest sensitivity among all serological tests [1] and this was confirmed by the results obtained by Shaapan *et al.* [19].

In view of the inadequate epidemiological data on toxoplasmosis in small ruminant slaughtered for human consumption, economic significance of small ruminants and their potential to spread toxoplasmosis to humans in Ethiopia, the present study was initiated with the objectives of estimating the seroprevalence and risk factors of *T. gondii* infection in sheep and goats slaughtered for human consumption in Debre-Zeit export abattoir.

## Methods

### Study animals

The study was conducted in sheep and goats brought to an export abattoir in Debre-Zeit town from two purposively selected Zones of Central Ethiopia (East Shewa and West Shewa). The origin of the animals is mainly from Fentale, Adea (East Shewa Zone) and Ambo (West Shewa Zone) districts. In this study, few females were sampled because females are normally kept for breeding purpose and those arriving at the abattoir are culls and/or infertile animals. Sheep and goats of different breeds aged over six months were included in this study. Age was determined by observation of the erupted permanent incisors [20]. Animals’ ≤ 1 year were considered as young while those above one year were considered adult.

### Study design and sample size

This cross-sectional study was carried out from September, 2011 to November, 2012. The numbers of study animals were determined based on an expected prevalence of 19.8% for goats [14], 31.6% for sheep [13], and 5% absolute precision using the formula described by Thrusfield [21]. Accordingly, the calculated sample sizes were 333 for sheep and 244 for goats (total = 577). However, 305 sheep and 323 goats (total = 628 animals) were randomly sampled.

### Blood collection

Blood samples were collected directly from heart chambers after slaughtering, properly labeled and the sera were separated by centrifugation at 3200 RPM for 10 minutes. The extracted sera were transferred to other sterile vials and kept at −20°C until serologically assayed.

### Serological examination

*Toxoplasma gondii*-specific IgG antibodies were detected by the direct agglutination test (DAT) (Toxo screen DA, biomerieux®, France) following the procedure described by manufacturer of the kit. Sera were assayed at a screening dilution of 1/40 and 1/4000 in order to avoid the false negative results that might happen at low dilutions when using sera with high antibody titers. A titer of 1: 40 or 1: 4000 or both was considered indicative of *T. gondii* exposure. Sedimentation of antigen at the bottom of the well and clear agglutination above half of the well at either dilutions were recorded as negative and positive results respectively. Positive and negative controls were included in each test. DAT positive samples were titrated to know the endpoint titer.

### Statistical analyses

The data generated were stored in Microsoft excel spreadsheet (Microsoft Corporation) and analyzed using STATA version 11.0 for Windows (Stata Corp. College Station, USA). Continuous data collected from abattoir for age was later on changed to categorical data for ease of handling. All the variables assessed were categorical and variables with more than two categories were transformed into indicator (dummy) variables. Seroprevalence was calculated by dividing the number of animals tested positive by the total number of animals tested. Association of risk factors (independent variables) with dependent variable (*T. gondii* positivity) was initially assessed using cross tabulation. Univariable logistic regression analysis was performed, and odds ratios (OR) and 95% confidence intervals (CI 95%) were used to quantify the strength of association between risk factors and *T. gondii* infection. Collinearity was assessed using collinearity matrix but none of the variables were found collinear. Variables that presented *P*-value of < 0.20 in univariable analysis were entered in the multivariable logistic regression model. The 95% confidence level was used and results were considered significant at *P* ≤ 0.05.

### Ethics statement

This research project was reviewed and approved by the ethical committee for animal experimentation of the College of Veterinary Medicine and Agriculture, Addis Ababa University, Ethiopia. All animals were handled in strict accordance with good animal practice.

## Results

### Overall seroprevalence

The overall seroprevalence of anti-*Toxoplasma gondii* IgG antibodies in small ruminants was 17.68% (95% confidence interval [CI]:14.77 − 20.89). Seroprevalence of 15.48% in goats (95% CI: 11.71 − 19.89) and 20% (95% CI: 15.6 − 24.94) in sheep was found. The highest seropositivity was recorded from sheep of West Shewa Zone and from goats originated from East Shewa Zone (Table 1).

**Table 1:** **Overall IgG seroprevalence of** ***T. gondii*** **infection in sheep and goats according to study area**

Study zones	Sheep		Goats		Total	
	No. tested	No. positive (%)	No. tested	No. positive (%)	No. tested	No. positive (%)
East Shewa	210	33 (15.71)	233	38 (16.31)	443	71 (16.03)
West Shewa	95	28 (29.47)	90	12 (13.33)	185	40 (21.62)
Total	305	61 (20.00)^a^	323	50 (15.48)^b^	628	111 (17.68)^c^

^a^significant between zones (*P* < 0.05), ^b^not significant (*P* > 0.05), ^c^not significant (*P* > 0.05).

The number of sheep and goats sera samples examined serologically using DAT, become positive from each study zones along with the total number of small ruminants tested and become positive.

Thirty-four per cent of DAT positive goats have end titer of ≥6000 (≥600 IU/ml) while 60.7% of seropositive sheep have end titer of ≥ 6000 (≥600 IU/ml). The DAT end titer of animals found positive for the screening was depicted in Table 2.

**Table 2:** **DAT end titers of seropositive sheep and goats samples**

Species	No. tested	No. positive	Reciprocal DAT titers							
			Positive at 1:40				Positive at 1:4000			
			60	180	540	1620	6000	18000	540000	162000
Sheep	305	61	14	3	2	5	9	6	11	11
Goat	323	50	18	5	3	7	4	4	5	4
Total	628	111	32	8	5	12	13	10	16	15

Note: According to the WHO third international standard preparation reciprocal antibody titer of 10 is equivalent to 1 IU/ml, i.e., 1:180 is equivalent to 18 IU/ml.

Number of sheep and goats sera tested and number positive at screening dilutions (1:40 and 1:4000) along with DAT end titer. The numbers under each dilution indicate the number of sheep or goat samples positive at that serum dilution.

### Risk factors

#### Goats

Univariable analysis of the association between putative risk factors and *T. gondii* seropositivity in goats showed that all variables considered have no statistically significant association (*P* > 0.05).

#### Sheep

The seroprevalence of toxoplasmosis was significantly different between the origins of sheep in that it was higher in sheep from West Shewa Zone (29.47%) than sheep from East Shewa Zone (15.71%) (χ2 = 7.7397; *P* = 0.005; Table 1). Univariable analysis of the association between putative risk factors and *T. gondii* seropositivity showed that sheep from West Shewa are 2.24 times more likely to be seropositive than sheep from East Shewa (*P* ≤ 0.05). In sheep, seroprevalence is higher in Horro breed than Afar breed and in dry season than wet season.

None of the factors investigated in goat are independent predictors of *T. gondii* infection while season and age were found to be independent predictors of seropositivity in sheep (Table 3).

**Table 3:** **Results of multivariable logistic regression analysis of risk factors and their association with** ***T. gondii*** **seropositivity**

Risk factors ^a^	Category		Adjusted OR ^b^ (95% CI)	P-***value***
		**Goat**		
Breed	Arsi-Bale		1.00	-
	Western highland		1.13 (0.48, 2.66)	0.772
	Afar		1.73 (0.83, 3.59)	0.141
Season	Wet		1.00	-
	Dry		1.64 (0.82, 3.27)	0.164
		**Sheep**		
Origin	East Shewa		1.00	-
	West Shewa		1.69 (0.75, 3.78)	0.203
Sex	Male		1.00	-
	Female		3.14 (0.80, 12.43)	0.102
Age	Young		1.00	-
	Adult		2.02 (1.10, 3.70)	0.**023**
Breed	Afar		1.00	-
	Arsi-Bale		2.64 (0.89, 7.78)	0.079
Season	Wet		1.00	-
	Dry		4.19 (1.55, 11.33)	0.**005**

^a^The variables included to the multivariable model were those with *P* ≤ 0.20 in the univariable analysis; ^b^Odds ratio adjusted for the variables in the table, Horro breed of sheep was dropped due to collinearity with origin. The bold P-values are significant.

Factors associated with *T. gondii* seropositivity in study zones with their category, adjusted odds ratio (aOR) and corresponding 95% confidence interval (95% CI) in the final multivariable logistic regression model.

## Discussion

### Overall seroprevalence

In the present study*,* the seroprevalence recorded in sheep (20.0%) was found to be relatively higher than in goats (15.48%). This is similar to the previous reports from Ethiopia [11, 13–15] and Ghana [22]. This might be due to the differences in feeding habits since sheep are more likely to get infection from the pasture as they graze close to the ground than goats which prefer browsing. Higher percentage of seropositive sheep (60.7%) had also end titer of ≥600 IU/ml compared to goats (34.0%) perhaps due to more frequent infection or re-infection by the parasite in the environment. The seroprevalence recorded in sheep is in close agreement with the seroprevalence estimated in previous studies in Ethiopia [10] but lower compared to seroprevalence reports of various investigators [13, 23–27]. Much higher seroprevalence in sheep than the present finding has been reported [4, 11, 15, 28–32].

The seroprevalence recorded in goats (15.48%) from Central Ethiopia was consistent with the result of Kassali and Teklye [10], Bisson *et al*. [33], Jittapalapong *et al*. [34], Ramzan *et al*. [27] and Zewdu *et al.* [14]. However, our result is lower than the seroprevalence reported by Demissie and Tilahun [24], Negash *et al.* [11], Hove *et al*. [28], Teshale *et al*. [12], Yibeltal [15] and Barakat *et al*. [35].

The differences in the prevalence observed between the present study and aforementioned studies could be due to differences in the relative cat densities, the access of sheep and goats to contaminated feed and water, the agro-climatic variation, the age and sex of the animals examined, the diagnostic techniques used [36, 37], cut-off value and sample size [38]. The high seroprevalence in the current study could be ascribed to suitable climatic conditions, wide spread presence of cats and extensive animal production systems [1, 4, 13, 14]. In the present study, identification of *T. gondii* seropositive sheep and goats was done using DAT due to its high sensitivity and specificity among all serological tests. Moreover, DAT is a major recommended test for diagnosis of toxoplasmosis in numerous species of animals and humans [1].

### Risk factors

This study used samples from abattoir where the majority of the slaughtered animals are young (55.7%, 350/628) and male (95.9%, 602/638). The significantly high prevalence in adult sheep than young sheep is due to high chance of exposure to the source of infection as the age increases and suggests that most sheep acquire the infection post-natal [1, 13, 15, 27].

Sheep sampled during the dry season (December to March) have four time more chance of seropositivity (*P* = 0.005) as compared to those sampled during wet season (April to November). This might be a reflection of fluctuations or differences in rate of transmission between seasons in that more infections taking place in the preceding wet months (where the climate is more suitable for survival of the oocysts) were being carried to dry season. Since IgG antibodies to *T. gondii* are long lasting in the body of the animals, the high positivity seen in dry season might partly be due to the carry over effect from preceding wet season infections (i.e. it doesn’t necessarily mean that serpositive animals sampled in dry season are infected in same dry season).

Univariable logistic regression analysis showed that seroprevalence in sheep was significantly associated with study areas and breed of sheep (*P* < 0.05). However, these variables were not found to be independent predictors of seropositivity in the multivariable model. Seroprevalence in sheep of West Shewa Zone (29.47%) was significantly higher than East Shewa Zone (15.71%) (*P* = 0.006). This might be due to climatic differences between the two Zones. Most areas of East Shewa Zone are low land with arid and semi-arid conditions less suitable for survival of oocysts of *T. gondii* in the external environment [13, 14]. Thus, the tenacity and infectivity of oocysts in East Shewa Zone might be less compared to West Shewa Zone. Horro breed of sheep were twice more likely to be seropositive (OR = 2.16, 95% CI: 1.01-4.61; *P* = 0.047) compared to Afar breed of sheep. Breed difference in *T. gondii* seropositivity was unknown.

In the present study, the presence of antibodies in sheep and goats demonstrate an exposure to *T. gondii* but does not confirm or necessarily indicate that these animals are currently sources of meat borne infection to humans. This is due to the fact that some seropositive small ruminants might harbor non-viable tissue cysts. However, considerable numbers of these seropositive animals were found to harbor viable tissue cysts of *T. gondii* in edible organs (Gebremedhin *et al*., unpublished) thus posing potential threat to consumers. The widespread exposure of small ruminants to oocysts of *T. gondii* in the present study corroborates well with the high seroprevalence (81.4%) of toxoplasmosis in women of child-bearing age from the same study Zones of Central Ethiopia reported recently [39].

The over representation of male and young animals included in the present study, due to higher presentation of such groups of food animals for slaughter and subsequent export to Arabian countries, might introduce some bias in underestimating the overall seroprevalence estimate of the population of origin. Hence, the information might not be inferable to the general population. Nevertheless, the prevalence estimate could be used for assessment of risk of small ruminant meat for people.

## Conclusions

The seroprevalence of *T. gondii* infection in small ruminants slaughtered for human consumption in Central Ethiopia is high. Age and season are significant predictors of seropositivity in sheep. The study highlighted the potential public health importance of meat of sheep and goats as source of infection for humans. Such prevalence studies can contribute in risk assessment of small ruminant’s meat for human consumption. Prevention of the spread of the disease through farm biosecurity measures is essential.
